# RETRACTION: Comparison of Clinical Outcomes Between Intravascular Ultrasound‐Guided and Angiography‐Guided Drug‐Eluting Stent Implantation: A Meta‐Analysis of Randomised Control Trials and Systematic Review

**DOI:** 10.1111/iwj.70631

**Published:** 2025-04-23

## Abstract

**RETRACTION:** TanY.Y. , ManX.X. , LiuL.Y. , and XuH. , “Comparison of Clinical Outcomes Between Intravascular Ultrasound‐Guided and Angiography‐Guided Drug‐Eluting Stent Implantation: A Meta‐Analysis of Randomised Control Trials and Systematic Review,” International Wound Journal 16, no. 3 (2019): 649–658, 10.1111/iwj.13073.30697972 PMC7949287

The above article, published online on 30 January 2019, in Wiley Online Library (http://onlinelibrary.wiley.com/), has been retracted by agreement between the journal Editor in Chief, Professor Keith Harding; and John Wiley & Sons Ltd. Following an investigation by the publisher, all parties have concluded that this article was accepted solely on the basis of a compromised peer review process. The editors have therefore decided to retract the article. The authors did not respond to our notice regarding the retraction.

**RETRACTION:** Y.Y. Tan , X.X. Man , L.Y. Liu , and H. Xu , “Comparison of Clinical Outcomes Between Intravascular Ultrasound‐Guided and Angiography‐Guided Drug‐Eluting Stent Implantation: A Meta‐Analysis of Randomised Control Trials and Systematic Review,” International Wound Journal 16, no. 3 (2019): 649–658, 10.1111/iwj.13073.30697972 PMC7949287

The above article, published online on 30 January 2019, in Wiley Online Library (http://onlinelibrary.wiley.com/), has been retracted by agreement between the journal Editor in Chief, Professor Keith Harding; and John Wiley & Sons Ltd. Following an investigation by the publisher, all parties have concluded that this article was accepted solely on the basis of a compromised peer review process. The editors have therefore decided to retract the article. The authors did not respond to our notice regarding the retraction.

